# Exploring the neural and behavioral correlates of cognitive telerehabilitation in mild cognitive impairment with three distinct approaches

**DOI:** 10.3389/fnagi.2024.1425784

**Published:** 2024-06-27

**Authors:** Silvia Paola Caminiti, Sara Bernini, Sara Bottiroli, Micaela Mitolo, Riccardo Manca, Valentina Grillo, Micol Avenali, Roberto De Icco, Sabina Capellari, Giovanni Augusto Carlesimo, Annalena Venneri, Cristina Tassorelli

**Affiliations:** ^1^Department of Brain and Behavioral Sciences, University of Pavia, Pavia, Italy; ^2^IRCCS Mondino Foundation, Pavia, Italy; ^3^Department of Medicine and Surgery, University of Parma, Parma, Italy; ^4^IRCCS Istituto delle Scienze Neurologiche di Bologna, Bologna, Italy; ^5^Department of Life Sciences, Brunel University London, Uxbridge, United Kingdom; ^6^Department of Biomedical and Neuromotor Sciences, University of Bologna, Bologna, Italy; ^7^Department of Systems Medicine, Tor Vergata University, Rome, Italy; ^8^IRCCS S. Lucia Foundation, Rome, Italy

**Keywords:** telerehabilitation, cognition, brain connectivity, non-pharmacological interventions, neurodegenerative diseases, cognitive training

## Abstract

**Background:**

Currently, the impact of drug therapies on neurodegenerative conditions is limited. Therefore, there is a strong clinical interest in non-pharmacological interventions aimed at preserving functionality, delaying disease progression, reducing disability, and improving quality of life for both patients and their caregivers. This longitudinal multicenter Randomized Controlled Trial (RCT) applies three innovative cognitive telerehabilitation (TR) methods to evaluate their impact on brain functional connectivity reconfigurations and on the overall level of cognitive and everyday functions.

**Methods:**

We will include 110 participants with mild cognitive impairment (MCI). Fifty-five participants will be randomly assigned to the intervention group who will receive cognitive TR via three approaches, namely: (a) Network-based Cognitive Training (NBCT), (b) Home-based Cognitive Rehabilitation (HomeCoRe), or (c) Semantic Memory Rehabilitation Training (SMRT). The control group (*n* = 55) will receive an unstructured home-based cognitive stimulation. The rehabilitative program will last either 4 (NBTC) or 6 weeks (HomeCoRe and SMRT), and the control condition will be adapted to each TR intervention. The effects of TR will be tested in terms of Δ connectivity change, obtained from high-density electroencephalogram (HD-EEG) or functional magnetic resonance imaging at rest (rs-fMRI), acquired before (T0) and after (T1) the intervention. All participants will undergo a comprehensive neuropsychological assessment at four time-points: baseline (T0), within 2 weeks (T1), and after 6 (T2) and 12 months (T3) from the end of TR.

**Discussion:**

The results of this RCT will identify a potential association between improvement in performance induced by individual cognitive TR approaches and modulation of resting-state brain connectivity. The knowledge gained with this study might foster the development of novel TR approaches underpinned by established neural mechanisms to be validated and implemented in clinical practice.

**Clinical trial registration:** [https://classic.clinicaltrials.gov/ct2/show/NCT06278818], identifier [NCT06278818].

## Introduction

Neurodegenerative diseases, such as Alzheimer’s disease (AD) and, in some cases Parkinson’s disease (PD), are characterized by progressive cognitive decline (Gonzales et al., 2022). Mild cognitive impairment (MCI) could represent a prodromal phase of different forms of dementia, in which, subjects present with objective and measurable cognitive deficits, although they maintain independence in daily-life activities (Yaffe et al., 2006).

No consensus has been reached on the effects of non-pharmacological interventions to contrast cognitive decline, but there is a general agreement that these approaches are more likely to succeed the sooner they are implemented and the more accurately they act on the cognitive domains affected by neurodegenerative diseases (Yao et al., 2020). Previous evidence showed effects of both single-domain and multi-domains cognitive rehabilitation on brain functionality in MCI (Rosen et al., 2011; Hampstead et al., 2012; Suo et al., 2016; Barban et al., 2017; De Marco et al., 2018).

Recently, several non-pharmacological interventions have been implemented in telerehabilitation (TR). TR is a promising approach as it offers and facilitates rehabilitative services to a broader population, and it has the potential to lower costs for both healthcare providers and patients (Kruse et al., 2020). However, there are still some aspects that need to be addressed in the implementation of TR for older adults with early cognitive deficits, including issues related to self-confidence, digital literacy, user experience, frequency of use, and dependence on guidance (Md Fadzil et al., 2022).

Randomized Controlled Trials (RCT) with adequate sample sizes and trial methodologies aimed at mitigating bias are necessary to assess the efficacy of TR for individuals at risk of dementia.

Specifically, in the current longitudinal multicenter RCT, we will apply three different cognitive TR approaches to patients with MCI due to neurodegenerative diseases. This clinical trial protocol aims to establish the feasibility and efficacy of three different cognitive TR approaches, and their impact on brain functional connectivity/synchronization, assessed by means of either high-density electroencephalogram (HD-EEG) or functional magnetic resonance imaging at rest (rs-fMRI).

The first TR approach is the Network-based Cognitive Training (NBCT), a cognitive training specifically designed to promote the co-activation of multiple brain areas (i.e., central nodes), modulating the functional connectivity of specific resting state networks (RSNs). This rehabilitation package has been used in face-to-face mode on a sample of healthy participants, in whom a rehabilitation-induced “up-regulation” effect of functional connectivity of the rear central nodes of the default mode network (DMN) was observed (De Marco et al., 2017). Subsequently, this training approach has been tested in amnestic MCI (De Marco et al., 2018), PD with MCI (Venneri et al., 2021) and in patients with relapsing–remitting multiple sclerosis who complained of mild cognitive deficits (Manca et al., 2021). Notably, the NBCT program will be implemented on a TR platform (i.e., Khymeia) and can be administered through a virtual connection. A link will be provided for each rehabilitation session, and this allows participants to access and use the TR protocol through a web browser on their personal computer, tablet, or mobile phone. This approach offers many advantages in terms of practicality, ease of use and accessibility for study participants.

The second TR approach is the Home-based Cognitive Rehabilitation (HomeCoRe) system. This is a software for cognitive TR especially developed for the initial stages of decline (amnestic MCI, MCI-PD, mild AD). HomeCore has been developed starting from a validated in-person computer-based cognitive intervention (CoRe) the efficacy of which in the short and long term has been verified in outpatients’ hospital services also in combination with neuromodulatory techniques (Alloni et al., 2017, 2018; Bernini et al., 2019, 2021, 2023; Rodella et al., 2022). HomeCoRe is an adaptive patient-tailored treatment focused on training memory and logical-executive functions that provides a Weighted Score of performance for each exercise and each session to monitor patients’ progress remotely and to adjust automatically the level of exercise difficulty accordingly. This strategy is applied to avoid either over- or under-stimulating patients. The software provides an easy-to -access platform that patients interact with via a touch screen. Usability and acceptance of HomeCoRe have already been tested in a small group of patients with MCI due to a neurodegenerative disease (Bernini et al., 2023).

The third TR approach is the Semantic Memory Rehabilitation Training (SMRT). This approach has been developed considering the crucial role that processing of semantic aspects of episodic information plays in long-term episodic memory processes (Craik and Lockhart, 1972), and based on recent evidence indicating early involvement of semantic memory in preclinical forms of Alzheimer’s disease (Wright et al., 2022). MCI patients will be trained to generate as many semantic features as possible they can in response to the visual presentation of objects from multiple semantic categories. The dual objective of this approach is to improve semantic memory processes and, capitalizing upon the richer semantic encoding of episodic events, make the retrieval processes more efficient. The protocol is administered through TR using a specific device with constant assistance from an online therapist.

## Methods and analysis

### Study design

**Figure 1:**
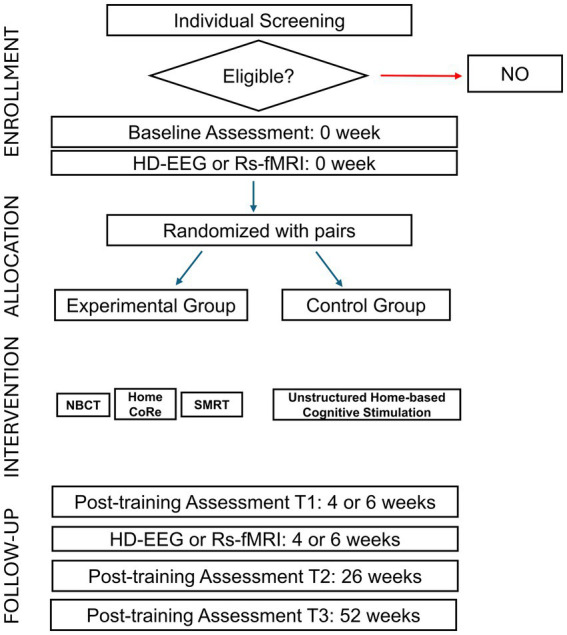
CONSORT flow chart for enrolment and randomization.

The proposed study is a prospective single-blind RCT, involving different Italian centers (Università di Pavia, AUSL Azienda Ospedaliero Universitaria di Parma, IRCCS Fondazione Santa Lucia di Roma, and IRCCS Istituto delle Scienze Neurologiche di Bologna). A CONSORT flow chart for enrolment and randomization is shown in Figure 1. After recruitment, participants will be contacted and will undergo in-person baseline assessment (T0) using the below-listed tests (see Participants’ evaluation section and Table 1). Participants who meet inclusion criteria will be enrolled and randomized to the active or control interventions (Table 1).

**Table 1:** Characteristics of included interventions.

Approach	Network-Based Cognitive Training (NBCT)	Home-based Cognitive Rehabilitation (HomeCoRe)	Semantic Memory Rehabilitation Training (SMRT)	Unstructured Home-based Cognitive Stimulation (Sham Comparator)
Participants	40 participants with MCI randomly allocated to NBCT.	5 participants with MCI randomly allocated to HomeCoRe.	10 participants with MCI randomly allocated to SMRT.	55 MCI, randomly assigned to one of three control groups (CG): CG for NBCT (*n* = 40), CG for HomeCoRe (*n* = 5), CG for SMRT (*n* = 10).
Interventions	Cognitive training specifically designed to promote cognitive functions in early stages of neurodegenerative conditions.	Cognitive training specifically designed to promote cognitive functions in early stages of neurodegenerative conditions.	Cognitive training specifically designed to promote cognitive functions in early stages of neurodegenerative conditions.	This activity is commonly used as a control condition to test the efficacy of innovative rehabilitative treatments.
Outcome measures	Primary outcome: Assessment of resting-state functional connectivity at baseline (T0) and at the end of NBCT (T1 after 4 weeks). Secondary outcome: Global cognitive and neuropsychological changes at T1, T2, and T3 vs. T0 when applicable according to Table 1. Secondary outcome measures will also include participant-centered assessment at T1.	Primary outcome: Assessment of resting-state functional connectivity at baseline (T0) and at the end of HomeCoRe (T1 after 6 weeks). Secondary outcome: Global cognitive and neuropsychological changes at T1, T2, and T3 vs. T0 when applicable according to Table 1. Secondary outcome measures will also include participant-centered assessment at T1.	Primary outcome: Assessment of resting-state functional connectivity at baseline (T0) and at the end of SMRT (T1 after 6 weeks). Secondary outcome: Global cognitive and neuropsychological changes at T1, T2, and T3 vs. T0 when applicable according to Table 1. Secondary outcome measures will also include participant-centered assessment at T1.	Primary outcome: Assessment of resting-state functional connectivity at baseline (T0) and at the end of sham intervention (T1 after 4 or 6 weeks). Secondary outcome: Global cognitive and neuropsychological changes at T1, T2, and T3 vs. T0 when applicable according to Table 1. Secondary outcome measures will also include participant-centered assessment at T1.

The primary active condition involves administering the NBCT protocol to 40 subjects with MCI across all included centers. The HomeCoRe protocol, involving 5 subjects, will be conducted exclusively in Pavia, while the SMRT protocol, involving 10 subjects, will be implemented only in Rome. The control condition consists in an unstructured home-based cognitive stimulation, involving 60 min of daily activities, with MCI sample size, frequency and overall treatment duration adapted to the experimental treatment of comparison. For each session, newspaper articles will be provided for the patient to read and summarize in a diary by answering specific questions. The protocol is administered remotely through TR with online assistance from a therapist.

Each patient will undergo brain rs-fMRI or HD-EEG acquisitions before (T0) and after (T1) active or control interventions.

Follow-up in-person neuropsychological assessments will be scheduled within 2 weeks from the end of the rehabilitation program (T1), and after 6 (T2) and 12 months (T3).

### Control for confounding variables and biases

After baseline assessment, patients included in the study will be randomly allocated to two groups in a ratio of 1:1 based on active and control interventions, controlling for sex and education levels. We will generate random numbers with a computer algorithm^1^ from a uniform distribution in the range 0–1, dividing the range into two equal intervals and assigning each participant to the group corresponding to the sampled number (1,1 ratio). Stratification will be employed to ensure equal distribution of participants within each stratum (e.g., blocks based on sex and education level), and block randomization will be used within each block. Propensity score matching will be applied using logistic regression and matching participants based on similar scores, to ensure comparable groups.

Neuropsychologists carrying out cognitive evaluations will be blinded to group allocation to prevent bias. They will receive appropriate instruction and guidance regarding all assessment procedures and outcome parameters. Reminders (e.g., written reminders, phone calls, and email messages) for each visit will be sent to all participants to ensure compliance and complete data collection.

Not all outcome measures will be administered at each time point (summary in Table 2).

**Table 2:** Evaluation battery across testing sessions.

	T0	T1	T2	T3
**Neuropsychological assessment**				
*Global cognition*				
Mini Mental State Examination (MMSE)	x	x	x	x
Montreal Cognitive Assessment (MoCA)	x	x	x	x
*Episodic long-term memory*				
Logical Memory Test immediate and delayed recall	x	x	x	x
Rey’s 15 words test immediate and delayed recall	x	x	x	x
Rey Complex Figure delayed recall	x	x	x	x
*Logical-executive functions*				
Rey Complex Figure copy	x	x	x	x
Raven’s Matrices 1947	x	x	x	x
Frontal Assessment Battery (FAB)	x	x	x	x
Semantic fluency	x	x	x	x
Phonological fluency (FAS)	x	x	x	x
*Working memory*				
Digit Span (forward/backward)	x	x	x	x
Corsi’s block-tapping test span (forward/backward)	x	x	x	x
*Attention/processing speed*				
Attentive Matrices	x	x	x	x
Trail Making Test A and B (TMT)	x	x	x	x
**Questionnaires and scales**				
*Functional level*				
Activities of Daily Living (ADL)	x			x
Instrumental Activities of Daily Living (IADL)	x			x
*Depressive symptoms*				
Beck Depression Inventory (BDI)	x	x	x	x
*Health status*				
12-Item Short Form Health Survey questionnaire (SF-12)	x	x	x	x
*Cognitive reserve*				
Cognitive Reserve Index questionnaire (CRIq)	x			
*Caregiver distress*				
Zarit Burden Inventory (ZBI)	x	x		
**Participant-centered outcomes**				
*Impression of symptom change*				
Patient Global Impression of Change (PGIC)		x		
*Treatment adherence*				
Number of sessions carried out		x		

T0 = baseline assessment; T1 = post-intervention assessment; T2 = 6-month follow up; T3 = 12-month follow up.

### Data management

Study data will be recorded in the REDCap database in compliance with the General Data Protection Regulation (GDPR). All participants will be registered with an identification code. The database will be kept updated to reflect the participant’s status at each stage during the course of the study. The collected data, after scientific publication, will be deposited in dedicated repositories according to the good practice of data sharing.

### Participants and eligibility criteria

Participants will be recruited from the Cognitive Disorders and Dementia Centre outpatient services and Movement Disorders and Neurorehabilitation Unit of IRCCS Mondino Foundation (Pavia, Italy), from the Unit of Neurology, University-Hospital of Parma (Parma, Italy), from the Neurological Clinic and NEUROMET of the IRCCS Institute of Neurological Sciences of Bologna-University of Bologna and the IRCCS S. Lucia Foundation of Rome. These patients will be asked to participate as volunteers in the study. Following their consent, they will be screened for eligibility criteria through a clinician evaluation carried out by an expert neurologist.

The inclusion criteria for participants will be:

- Diagnosis of amnestic MCI (Petersen et al., 1999) or Parkinson’s disease with MCI (Litvan et al., 2011);
- Aged between 60 and 85 years;
- Years of education ≥5;
- Clinical Dementia Rating (CDR) (Hughes et al., 1982) score = 0.5.
- The exclusion criteria will be:
- Mini-Mental State Examination (MMSE) score < 20;
- Presence of cognitive impairment secondary to an acute or general medical disorder (e.g., brain trauma, small vessel disease, vascular impairment or tumor);
- Presence of severe neuropsychiatric conditions (e.g., mood and behavioral disorders);
- Presence of severe sensory disorder (e.g., deafness or blindness) or motor impairments that prevent trunk control and/or sitting position;
- Current cognitive treatments;
- Lack of family support.

### Participants’ evaluation

See Table 1 for the evaluation battery (neuropsychological assessment, questionnaires and scales, and participant-centered outcomes) across testing sessions. Each evaluating session will be about 90 min per participant and will be carried out in a hospital setting.

### Neuropsychological assessment

The cognitive assessment, performed by using neuropsychological tests standardized for the Italian population, will evaluate the following cognitive domains:

- Global cognition
- Mini Mental State Examination (MMSE) (Magni et al., 1996);
- Montreal Overall Cognitive Assessment (MoCA) (Conti et al., 2015);
- Episodic long-term memory
- Logical Memory Test for immediate and delayed recall (Novelli et al., 1986; Spinnler, 1987)
- Rey’s 15 words test for immediate and delayed recall (Carlesimo et al., 1996);
- Rey Complex Figure delayed recall (Caffarra et al., 2002);
- Logical-executive functions
- Raven’s Matrices 1947 (Carlesimo et al., 1996);
- Frontal Assessment Battery (Appollonio et al., 2005);
- Semantic fluency (Novelli et al., 1986);
- Phonological fluency (FAS) (Carlesimo et al., 1996);
- Visuo-constructive functions
- Rey Complex Figure copy (Caffarra et al., 2002);
- Working memory
- Digit Span (Forward/Backward) (Monaco et al., 2013);
- Corsi’s block-tapping test span (Forward/Backward) (Monaco et al., 2013);
- Attention/processing speed
- Digit Cancellation Test (Spinnler, 1987);
- Trail Making Test A and B (Giovagnoli et al., 1996).
- Parallel forms (i.e., alternative versions using similar material) will be applied for follow-up visits when available in order to avoid possible learning effect. All test scores will be corrected for age, sex, and education by using appropriate correction grids and compared with the values available for the Italian population.

### Questionnaires and scales

Additionally, we will administer the questionnaires and scales reported below to evaluate the following aspects:

- Functional level
- Activities of Daily Living (ADL) (Lawton and Brody, 1969);
- Instrumental Activities of Daily Living (IADL) (Lawton and Brody, 1969);
- Depressive symptoms
- Beck Depression Inventory (BDI) (Beck et al., 1996);
- Health status
- 12-Item Short Form Health Survey questionnaire (SF-12)4 (Ware et al., 1996)
- Cognitive reserve
- Cognitive Reserve Index questionnaire (CRIq) (Nucci et al., 2012);
- Caregiver distress
- Zarit Burden Inventory (ZBI) (Nucci et al., 2012).

### Participant-centered outcomes for monitoring adherence to TR interventions

In order to assess the subjective evaluation of TR success, we will evaluate the following aspects at T1: (a) Impression of symptom change through the Patient Global Impression of Change (PGIC) (Hurst and Bolton, 2004). (b) Treatment adherence considering the number of sessions carried out by the patient. To monitor and report adherence accurately, we will implement several strategies.

We will use digital tracking tools integrated into the TR platform to log each session automatically. These tools will record the duration and completion status of each session, providing real-time data on patient adherence. Additionally, patients will complete adherence logs, which will be cross-referenced with the digital records to ensure accuracy.

To manage deviations from the planned protocol, we will establish predefined criteria for non-adherence (e.g., missing a specified number of sessions) and implement a follow-up protocol. This protocol will include automated reminders sent via email to encourage session completion and regular check-ins by a designated adherence coordinator who will contact patients directly to address any barriers to adherence and provide support.

Non-adherence will be documented, and reasons for missed sessions will be categorized (e.g., technical issues, personal reasons) to identify common obstacles and improve the TR program. Adherence data will be reported in both aggregate and individual formats, highlighting overall adherence rates, trends over time, and any significant deviations from the protocol. This detailed reporting will allow us to evaluate the effectiveness of our adherence strategies and make necessary adjustments to enhance patient engagement.

By implementing these comprehensive monitoring and management strategies, we aim to ensure high adherence rates and the successful delivery of the telerehabilitation interventions.

In order to assess subjective evaluation of TR success, we will evaluate the following aspects at T1: (a) Impression of symptom change through the Patient Global Impression of Change (PGIC) (Hurst and Bolton, 2004). (b) Treatment adherence considering the number of sessions carried out by the patient.

### Brain resting state connectivity measures

#### Rs-fMRI functional connectivity

Each center is equipped with a high field MRI scanner (i.e., GE or Siemens 3 T). A harmonized MRI protocol, already developed by the Neuroscience and Neurorehabilitation Network (RIN), the Italian largest research network in the neuroscience field, will be used. The protocol will include the following sequences: 3D T1 weighted images (3D T1w), 3D T2 weighted Fluid Attenuated Inversion Recovery (3D T2-FLAIR) images and resting-state functional magnetic resonance imaging (rs-fMRI).

Structural MRI images will be acquired using a 3D MPRAGE T1-weighted sequence with the following parameters: TR = 2,300 ms; TE = 2.98 ms; TI = 900 ms; flip angle = 9°; voxel size = 1.1 × 1.1 × 1.2 mm^3^; Field of view = 256 × 240 mm^2^; 170 slices. Resting-state fMRI images will be acquired with eyes closed using a T2-weighted echo-planar imaging sequence with the following parameters: TR = 3,000 ms; TE = 30 ms; flip angle = 80°; 48 slices of 3.3 mm; 140 volumes; acquisition duration ~10 min. Structural and functional MRI data will be pre-processed and analyzed using SPM12 (Wellcome Centre for Human Neuroimaging, London, United Kingdom) and the CONN toolbox. The following pre-processing steps will be applied: (1) slice-timing and realignment; (2) co-registration of structural and functional images; (3) segmentation of T1-weighted images into gray matter (GM), white matter (WM) and cerebrospinal fluid (CSF) tissue maps; (4) normalization of both T1- and T2*-weighted scans into the MNI space; and (5) smoothing of both images via a Gaussian kernel of 6 mm. Multiple denoising steps will be carried out on pre-processed T2*-weighted images: (1) regressing out the first 5 components of WM and CSF signal; (2) regressing out motion parameters; (3) application of a band-pass filter (0.008–0.1 Hz) to remove non-neural signals; (4) linear detrending; and (5) de-spiking. GM, WM and CSF volumes will be extracted and summed to calculate total intracranial volume for each participant to be used as a covariate in the analyses.

Seed-based analysis will be conducted on each patient’s rs-fMRI acquisitions to determine the pairwise correlation between the hemodynamic signals extracted from seed regions and the whole-brain signals at voxel level. The resulting maps are typically interpreted as representing the functional connectivity of those seed regions (Smith et al., 2014).

#### HD-EEG synchronous activity

HD-EEG will be acquired in the different centers following the same procedure. In details, signals will be acquired for 12 min (6 min eyes open and 6 min eyes closed) at a sampling frequency of 1,000 Hz. For HD-EEG recording, a HydroCel Geodesic Sensor NetTM cap will be utilized. The pre-wired cap allows for the correct placement of 128 electrodes with approximate inter-electrode distances of 20–25 mm. HD-EEG pre-processing and source localization: The HD-EEG will be recorded with a Notch filter centered at 50 Hz to eliminate related artifacts. EEG signals will be filtered (1–80 Hz) with a zero-phase distortion FIR filter and downsampled to 250 Hz. Biologically originated artifacts will be discarded using independent component analysis. An anatomical head model has been utilized, identifying 12 different tissue classes (skin, eyes, muscles, fat, spongy bone, compact bone, gray matter, cerebellar gray matter, white matter, cerebellar white matter, cerebrospinal fluid, and brainstem). Source reconstruction will be performed using exact Low-Resolution Brain Electromagnetic Tomography (eLORETA). After cortical source reconstruction using the LORETA method, different frequencies will be decomposed using the Fourier transform as a mathematical operator.

Brain connectivity will be investigated across all frequency bands using a seed-based technique, calculating connectivity between each seed and all other brain voxels. A 2-s Hamming window, with 50% overlap between consecutive windows, will be used to reconstruct frequencies ranging from 1 to 80 Hz. EEG connectivity maps will be created by estimating the Pearson’s correlation coefficient between each seed time series using a standard MATLAB^®^-based code.

### Outcome measures

#### Primary outcome measures

Our primary goal is to understand how TR may affect RSNs communications in participants diagnosed with MCI due to a neurodegenerative disease. Primary outcomes will be measured through the assessment of changes in resting-state brain connectivity obtained from HD-EEG and rs-fMRI techniques. This would enable monitoring variations in resting-state connectivity involved during rehabilitative activities. Resting-state brain connectivity plays a critical role in deactivation during specific tasks, but this process becomes compromised with aging and progression toward neurodegenerative diseases. Consequently, interventions aimed at restoring brain connectivity have the potential to provide cognitive benefits in the early stages of the disease.

We plan to assess rs-fMRI FC and/or HD-EEG SA at two time points: before the TR (T0) and within 2 weeks of TR completion (T1). All the obtained measures will be compared with the results obtained from the MCI participants assigned to the control groups.

#### Secondary outcome measures

As the secondary outcome measures, we will consider the longitudinal changes in all the neuropsychological tests, questionnaires, and scales (T1, T2, and T3 vs. T0 when applicable according to Table 1). Secondary outcome measures will also include participant-centered outcomes to assess those aspects that are most important for the participants and the subjective evaluation of intervention success at T1. We will also correlate these findings with changes in FC/SA between T0 and T1. Furthermore, we will evaluate caregiver burden at different time points.

### Planned analysis

#### Primary outcome analysis plan

Brain connectivity measures will be extracted from both rs-fMRI and HD-EEG data. The RSNs will be constructed considering a predetermined set of seed regions (Franco et al., 2013). As we will, for the first time, investigate the efficacy of three distinct TR approaches and their associated neural correlates, we will examine brain connectivity across all major RSNs.

For rs-fMRI seed-based timecourses will be extracted from each seed region, and individual maps of functional connectivity will be computed modeling the linear association between the timecourse of the seed and the timecourse of each voxel within the identified RSNs. For HD-EEG data, brain connectivity will be extracted by assessing the weighted Phase Lag Index (wPLI) (Vinck et al., 2011) that measures the phase synchronization of two signals. For both methods, the strength of the relationship between nodes of RSNs will be quantified using seed-based analysis, estimating pairwise correlations between a seed region and all the other voxels across the brain.

We will assess if the TR intervention has effects on changes in functional connectivity between T0 and T1 and whether these effects are absent at T0 vs. T1 in the control stimulation. This approach enhances the validity of the analyses by specifically addressing the impact of the treatment on connectivity changes over time and comparing them with a control condition.

Using the formula (Δ connectivity change = T1 − T0), the changes in Pearson’s correlation coefficient transformed into z-scores will be computed for each participant within each network. A large Δ connectivity change will represent a higher correlation coefficient (stronger connectivity) at T1 than T0.

The effect of the cognitive training program on network connectivity will be tested using mixed-design full-factorial scripts modeling the condition-by-timepoint interaction (increases Δ connectivity change seen in the experimental condition net of the increases seen in the control condition). We will also test the “inverse interaction” contrast (exclusive increases in Δ connectivity change seen in the control condition) as a methodological control. The statistical model will be run independently for each considered TR approach (i.e., NBCT, HomeCoRe, and SMRT).

To account for the nested data structures (e.g., patients nested within different centers), we will employ multilevel modeling (MLM). MLM will allow us to partition the variance at different levels (e.g., within-patient, between-patient, and between-center) and accurately estimate the effects of the cognitive training program while accounting for potential clustering. This approach will help in managing the hierarchical nature of the data, improving the precision of our estimates.

Potential confounding variables (center of acquisition, MCI subtype, age, sex, functional activities, current pharmacological treatment, cognitive reserve) will be considered as covariates.

#### Secondary objective analysis plan

Differences in global cognitive and neuropsychological performance scores, as well as caregiver burden assessments will be independently assessed using a two-way repeated measures ANOVA model, including assessment times and the TR interventions (active vs. control) as factors. The interaction between time and type of TR intervention will be investigated. To explore the variability in the trajectories of global cognitive and neuropsychological performance scores, individual items will be independently assessed using repeated measures linear mixed models. The time elapsed between assessments will be used to estimate the monthly rate of change, considering the type of TR intervention (active vs. control) as a main effect.

Furthermore, a linear correlation analysis will be applied to test the relationship between changes in connectivity from T0 to T1 and cognitive change over time. As mentioned above, the analyses will be conducted separately for each type of treatment/outcome and individual performance parameter.

We will re-run all the aforementioned analyses, stratifying by MCI subtype (aMCI and PD-MCI), to evaluate the disease-specific effects of TR on brain connectivity.

### Power analysis

In line with the findings from De Marco et al. (2017), where increased DMN resting-state connectivity was observed in patients assigned to the experimental condition in a midline cluster extending to the precuneus and cuneus, we aim to detect similar changes in brain functional connectivity through our study. De Marco et al. reported decreased connectivity in the right and left parietal cortices for the control condition. The experimental condition showed an average increase of 0.18 z scores, while the control condition exhibited an average decrease of 0.63 z scores (mixed-design ANOVA’s F1, 35 = 15.08, *p* < 0.001). Based on these findings, we conducted a power analysis to determine the necessary sample size for detecting differences in functional connectivity changes between the experimental and control groups. Assuming a medium effect size (Cohen’s *d* = 0.5), a significance level of 0.05, and a power of 0.90, our analysis indicated that a total sample size of approximately 80 participants (40 per condition, evaluated at two time points for NBCT) would be sufficient to detect medium to large effects, ensuring robust and reliable results.

### Ethical issues

This trial will involve human participants, cognitive TR interventions, data collection, elaboration and abstraction used for the evaluation of two therapeutic options. In addition to ethical approval, all procedures and data management strategies have been approved by the Data Protection Officer of the University of Pavia who guarantees compliance with the GDPR (General data protection regulation). Information provided when acquiring informed consent from participants will be given in a language appropriate to the individual level of understanding. Participants will also be encouraged to ask questions before signing the informed consent form.

To the best of our estimation, TR interventions should not have any potential negative impact on the participant. Moreover, both HD-EEG and rs-fMRI are non-invasive techniques for the study of human brain function.

The investigators will communicate any possible, unforeseen, adverse event to the Ministry of Health. Regarding payment policies for participants, any compensation amount, method and timing of disbursement must be consistent with the laws, regulations, and guidelines of the region in which the study is conducted and must not improperly influence a participant’s decision to participate. This trial is a no profit study and, in Italy, the national legislation states that it is forbidden to offer or request any kind of financial benefit/incentive for participation in clinical experimental trials.

Since participants are expected to interact with a TR tool, one possible issue could be frustration in case of lack of ability to cope with that technology. However, this risk will be mitigated, before the beginning of TR treatment, thanks to specific training sessions on the use of this application that will be provided to participants (and possible caregivers). Moreover, the interface is fully compliant with the guidelines for human-computer interaction, to make the user interface as easy as possible.

### Data monitoring

A permanent monitoring will be done by the clinical team, with the supervision by the research team and the local Ethics Committee. All the possible adverse events will be communicated immediately to the Ethics Committee to determine trial’s modifications or cessation.

## Discussion

Studies suggest that cognitive decline affects approximately 25–50% of older adults living in the community (Jonker et al., 2000; Mewton et al., 2014; Rodakowski et al., 2015). This decline can impact the performance of daily activities, potentially impacting lives of affected individuals and their families. Unfortunately, cognitive decline goes often undetected until the development of dementia. Furthermore, the number of individuals living with dementia is projected to double by 2030. The economic burden associated with dementia has surpassed the combined costs of cancer and cardiovascular disease (Luengo-Fernandez et al., 2015). These challenges stemming from cognitive decline necessitate proactive measures to address the situation.

Among the possible proactive measures, non-pharmacological approaches have the advantage of avoiding adverse side effects, are expected to be simpler to put into practice and are generally preferred among older demographics. TR, which uses information and communication technology to connect patients and therapists, has being explored as a potential solution for addressing the suboptimal rehabilitation outcomes observed in individuals with MCI (Vannini et al., 2017; Brown et al., 2022). Several research teams are currently engaged in clinical trials aimed at assessing the feasibility of home-based physical rehabilitation for this population.

The primary objectives of this clinical trial protocol are to investigate and compare the feasibility and efficacy of three distinct cognitive TR interventions among individuals diagnosed with MCI. Our assessment of TR efficacy will be based on the evaluation of alterations in brain functional connectivity alongside clinical and neuropsychological metrics. We postulate that all modes of TR delivery will demonstrate efficacy relative to the control group. The outcomes of this investigation hold potential to advocate for the integration of remote rehabilitation services. Moreover, this trial endeavors to contribute to the advancement of innovative TR methodologies through collaborative efforts within the telerehabilitation domain.

This study has some limitations that need to be acknowledged. Participants with limited computer skills and without a supportive caregiver might be excluded from using TR, introducing a selection bias in this type of intervention (Moo et al., 2020). Nevertheless, evidence suggests that telemedicine devices can be effectively used by individuals with early cognitive impairment who live alone (Smith et al., 2007). Furthermore, it should be noted that the three different TR interventions are characterized by a different overall duration and frequency of sessions, which could affect the comparability of the approaches. However, only maintaining the pre-existing characteristics of each intervention, we are able to establish their feasibility and efficacy, thus paving the way for larger confirmatory trials.

## Ethics statement

This study has been approved by the Local Ethics Committee and registered at https://clinicaltrials.gov (NCT06278818). The dissemination plan includes the scientific community through publication in open-access peer-reviewed scientific journals and presentations at national and international conferences.

## Author contributions

SPC: Conceptualization, Formal analysis, Methodology, Writing – original draft, Writing – review & editing. SBe: Conceptualization, Data curation, Methodology, Resources, Writing – original draft, Writing – review & editing. SBo: Conceptualization, Data curation, Methodology, Resources, Writing – original draft, Writing – review & editing. MM: Data curation, Writing – original draft, Writing – review & editing. RM: Data curation, Writing – original draft, Writing – review & editing. VG: Data curation, Writing – review & editing. MA: Data curation, Writing – review & editing. RDI: Data curation, Writing – review & editing. SC: Conceptualization, Funding acquisition, Writing – review & editing. GAC: Conceptualization, Funding acquisition, Writing – review & editing. AV: Funding acquisition, Writing – review & editing. CT: Funding acquisition, Writing – original draft, Writing – review & editing.

## Footnotes

^1^ https://www.random.org/
